# An improved method for precise genome editing in zebrafish using CRISPR-Cas9 technique

**DOI:** 10.1007/s11033-020-06125-8

**Published:** 2021-01-22

**Authors:** Eugene V. Gasanov, Justyna Jędrychowska, Michal Pastor, Malgorzata Wiweger, Axel Methner, Vladimir P. Korzh

**Affiliations:** 1 International Institute of Molecular and Cell Biology in Warsaw, Ks. Trojdena Str. 4, 02-109 Warsaw, Poland; 2 Postgraduate School of Molecular Medicine, Medical University of Warsaw, Zwirki i Wigury Str. 61, 02-091 Warsaw, Poland; 3 Institute of Biochemistry and Biophysics of Polish Academy of Sciences, Pawinskiego Str. 5a, 02-106 Warsaw, Poland; 4 Institute of Molecular Medicine, University Medical Center Mainz, Langenbeckstr. 1, 55131 Mainz, Germany

**Keywords:** CRISPR-Cas9, Zebrafish, Precise deletion editing, gRNAs, HRM

## Abstract

Current methods of CRISPR-Cas9-mediated site-specific mutagenesis create deletions and small insertions at the target site which are repaired by imprecise non-homologous end-joining. Targeting of the Cas9 nuclease relies on a short guide RNA (gRNA) corresponding to the genome sequence approximately at the intended site of intervention. We here propose an improved version of CRISPR-Cas9 genome editing that relies on two complementary guide RNAs instead of one. Two guide RNAs delimit the intervention site and allow the precise deletion of several nucleotides at the target site. As proof of concept, we generated heterozygous deletion mutants of the *kcng4b*, *gdap1*, and *ghitm* genes in the zebrafish *Danio rerio* using this method. A further analysis by high-resolution DNA melting demonstrated a high efficiency and a low background of unpredicted mutations. The use of two complementary gRNAs improves CRISPR-Cas9 specificity and allows the creation of predictable and precise mutations in the genome of *D. rerio*.

**Supplementary Information:**

The online version contains supplementary material available at 10.1007/s11033-020-06125-8.

## Introduction

CRISPR-Cas9-based site-specific mutagenesis is more and more widespread in life sciences [1]. It is mainly used to inactivate (knock-out, KO) target genes by deletions and small insertions or to insert genes encoding marker proteins (knock-in, KI) at specific genomic sites of various model organisms [2]. The standard CRISPR-Cas9 KO procedure includes the design of a short guide RNA (gRNA) corresponding to the genome sequence at or close to the site of intervention and the injection of cells or developing embryos with this gRNA and Cas9 mRNA or protein. This then results in DNA double-strand breaks at the target site caused by the Cas9 nuclease and subsequent non-homologous DNA repair resulting in random mutations at the target site [2, 3]. The creation and injection of all CRISPR-Cas9 components (gRNAs and Cas9 mRNA) into zebrafish (*Danio rerio*) embryos is a rather simple and well-established procedure. At the same time, the screening for the desired mutants is time-consuming. Another disadvantage is the unpredictable nature of such mutagenesis. Several methods were proposed to simplify the mutagenesis and selection of mutants [3, 4]. These include the usage of more than one gRNA at once to delete large segments of DNA in animal cells [5], plants [6] and invertebrates, namely *Dictyostelium discoideum* [7], *Drosophila melanogaster* and *Caenorhabditis elegans* [8]. CRISPR-Cas9 with two gRNAs was also used to delete from 1000 to 20,000 nucleotide base pairs (bp) in zebrafish [9].

Here we describe a significant improvement of CRISPR-Cas9 mutagenesis which allows the precise deletion of several nucleotides using of two gRNAs per target site. As an efficient method for mutant screening we propose the PCR-based high-resolution DNA melting (HRM) analysis. Using this approach, we generated two short deletion mutants of *kcng4b* (α-subunit of the voltage-gated potassium channel), a deletion mutant of *gdap1* (ganglioside-induced differentiation-associated protein 1), and a deletion mutant of *ghitm* (growth hormone-inducible transmembrane protein).

## Materials and methods

### Animals

Wild-type (AB line) and mutant zebrafish were maintained in the Zebrafish Facility of the International Institute of Molecular and Cell Biology in Warsaw (license no. PL14656251) according to standard procedures and ethical regulations. All experiments involving the zebrafish embryos/larvae were carried out in accordance with the Polish Laboratory Animal Science Association and Local Ethics Committee for Animal Experimentation in Warsaw (permission no. WAW2/181/2019).

To perform CRISPR-Cas9 knock-out, 2 nl of a solution containing two gRNAs (0.04 mg/ml of each) and Cas9 protein (0.2 mg/ml, see below) were injected into the cytoplasm of one-cell stage wild-type AB zebrafish embryos. Fifty embryos of each, *gdap1* and *ghitm*, as well as two variants of *kcng4b* (variants #1 and #2) knock-outs were grown up after injection to 4 months to be in-crossed as the F0 founders. The phenotype of the F1 off-spring was analyzed at 1–5 dpf under a Leica M165 FC microscope. All F0 founders were fin-clipped for DNA sampling and HRM analysis. The mutation-positive ones were analyzed by DNA sequencing.

Standard single gRNA mutagenesis was performed as a control. For this, the 5′-site gRNAs of each set (all gRNAs-1 and Kcng4b-gRNA-3) were injected into 50 embryos as described above. The fish were analyzed by HRM at 5 days post fertilization (dpf) stage.

### gRNA synthesis

DNA oligonucleotides encoding gRNAs with invariant adapter sequence were used, four for *kcng4b* and two for *gdap1* and *ghitm*: Kcng4b-gRNA-1, TAATACGACTCACTATATGAAGAGAGACTCCTTTTCTGTTTTAGAGCTAGAA; Kcng4b-gRNA-2, TAATACGACTCACTATAGTTAGCAATGGCCCAAGAAAGTTTTAGAGCTAGAA; Kcng4b-gRNA-3, TAATACGACTCACTATAAGCAGTGAGGGTTGGCTGAAGTTTTAGAGCTAGAA; Kcng4b-gRNA-4, TAATACGACTCACTATACATGCAGCAGCAGTGAGGGTGTTTTAGAGCTAGAA; Gdap1-gRNA-1, TAATACGACTCACTATAGGGAGTCTACGGTGATCTCTGTTTTAGAGCTAGAA; Gdap1-gRNA-2, TAATACGACTCACTATAGGCTGGTATGTGGGAGTCTAGTTTTAGAGCTAGAA; Ghitm-gRNA-1, TAATACGACTCACTATACGCGGGCAGTGTGGGCCTGAGTTTTAGAGCTAGAA; Ghitm-gRNA-2, TAATACGACTCACTATATGTGGGCCTGACGGCGCTCTGTTTTAGAGCTAGAA (gRNA sequences are underlined, all sequences here and below have the 5′–3′ direction).

For gRNA synthesis each of them was mixed with the constant oligonucleotide, containing a complementary adapter part and a T7 promoter sequence, Const-gRNA: AAAAGCACCGACTCGGTGCCACTTTTTCAAGTTGATAACGGACTAGCCTTATTTTAACTTGCTATTTCTAGCTCTAAAAC, both 10 μM, and a double-stranded product was synthesized according to the following protocol: 95 °C—3 min and 35 cycles of 95 °C—15 s, 20 °C—15 s, 72 °C—15 s using PCR Mix Plus mixture (A&A Biotechnology, Poland). The resulting DNA was purified by Clean-Up Concentrator kit (A&A Biotechnology, Poland) and verified by 2.5% agarose gel-electrophoresis. 0.2 μg of DNA was used for RNA in vitro transcription by T7 RNA-Polymerase (A&A Biotechnology, Poland) according to the manufacturer’s protocol.

### Cas9 protein production

DNA encoding *Streptococcus pyogenes* Cas9 protein with N- and C-terminal nuclear localization signals (NLS) was PCR amplified using PCR Mix Plus mixture (A&A Biotechnology, Poland) and pCS2-nCas9n plasmid [11] as a template and cloned into pETM-60 vector (Novagen) using *Nco*I and *Xho*I restriction endonuclease sites. The final construct encoded an N-terminal hexa-histidine-NusA tag followed by peptide cleavage site for tobacco etch virus (TEV) protease and C-terminal hexa-histidine (His) tag. The protein was expressed in BL21 Rosetta *Escherichia coli* strain (Novagen) in simplified Studier’s autoinduction media [12] at 37 °C for 4 h followed by 20 h at 20 °C. Cells were harvested by centrifugation at 4000 × *g* for 30 min and stored at −20 °C.

The cell pellet was resuspended in 40 mM Tris–HCl buffer (pH 7.5) containing 5% of glycerol, 0.5 M NaCl, 40 mM imidazole, 1.0 mM phenylmethylsulfonyl fluoride (PMSF) and 2 mM 2-mercaptoethanol, then ultrasonicated and centrifugated at 10,000 × *g* 4 °C for 20 min. The supernatant was loaded on a HisTrap FF Crude^®^ column (GE Healthcare) equilibrated by the same buffer. After washing with 20 mM Tris–HCl buffer (pH 7.5) containing 5% of glycerol, 0.5 mM NaCl and 80 mM imidazole, the protein was eluted by the same buffer containing 0.3 M imidazole and the N-terminal His-NusA tag was cleaved off by TEV protease (1 mg per 100 mg of Cas9 protein) during overnight dialysis against 20 mM Tris–HCl buffer (pH 7.5) containing 5% of glycerol, 0.15 mM NaCl and 2 mM 2-mercaptoethanol. The protein solution was then clarified by centrifugation (10,000 × *g*, 10 min) and loaded on a Q Sepharose^®^ Fast Flow column (GE Healthcare) equilibrated by the same buffer. The flow-through was collected and dialyzed overnight against 20 mM HEPES buffer (pH 7.5) containing 0.2 mM KCl and 20% of glycerol. As a final step the protein sample was concentrated on a Vivaspin 500 centrifugal concentrator (Merk) and stored at −80 °C.

All Cas9 purification steps were controlled by spectrophotometry and by SDS-polyacrylamide gel electrophoresis.

### Fish mutation analysis

The clipped fins of adult fish or 5-day-old embryos chilled on ice were placed into 30 μl of 96% ethanol, heated at 80 °C until ethanol evaporates (~10 min) and 50 μl of TE buffer (pH 8.0) were added. After 10 min of heating (80 °C) samples were chilled and incubated 3 h at 55 °C with 0.5 mg/ml Proteinase K (Merck). After another heating (10 min, 95 °C) and chilling, 1 μl of the solution was used as a template for PCR and HRM analysis.

The HRM analysis was performed according to the manufacturers’ protocols (Roche and BioRad). PCR was performed in 10 μl volume with a 0.4 μM of each primer using Light Cycler 480 High-Resolution Melting Master Mix, 2 mM MgCl_2_ (Roche) or using Precision Melt Supermix (BioRad). Melting curves were taken by Light Cycler 96 (Roche) or by CFX Connect Real-Time PCR System (BioRad). The following oligonucleotides were used as the primers:

- For *kcng4b* #1: Kg4-HRM-1f, GATGCCACTGCTAAAGAGG, and Kg4-HRM-1r, GGTTGGCTGAATGGCAGC; and for *kcng4b* #2: Kg4-HRM-2f, TGCTAACAGAAATAGCCTGC, and Kg4-HRM-2r, CTCATTAAAACATACAGAAATCC.
- For *gdap1*: GDAP1-HRM-f, GGAAGCACGTATTATCATCG; and GDAP1-HRM-r, TGCGAATGTGTGTAGTGGC.
- For *ghitm*: GHITM-HRM-f, CGATCTGGCCGCAGTACG; and GHITM-HRM-r, TACCAGCCAGGAGTTGCTC.

The following PCR conditions were used: pre-incubation: 95 °C—10 min; 3 step amplification (45 cycles): 95 °C—10 s, 60 °C—15 s, 72 °C—15 s; high resolution melting: 95 °C—60 s, 40 °C—60 s, 65 °C—1 s, 97 °C—1 s.

For the PCR and following sequencing *kcng4b*-specific primers were used: Kcng4b-seq-fvd, CGTTCATATCACGAACTGAAG, and Kcng4b-seq-rev, GGTAGGTCAAATCTTTGAAAAC; while the *gdap1* and *ghitm* fragments were amplified using the HRM primers. The following PCR conditions were used: pre-incubation: 95 °C—3 min; 3 step amplification (35 cycles): 95 °C—30 s, 58 °C—30 s, 68 °C—15 s. PCR-products were got using PCR Mix Plus mixture from A&A Biotechnology (Poland) and sequenced from the same primers. Heterozygous sequences were analyzed using the TIDE web tool [10].

## Results

**Fig. 1:**
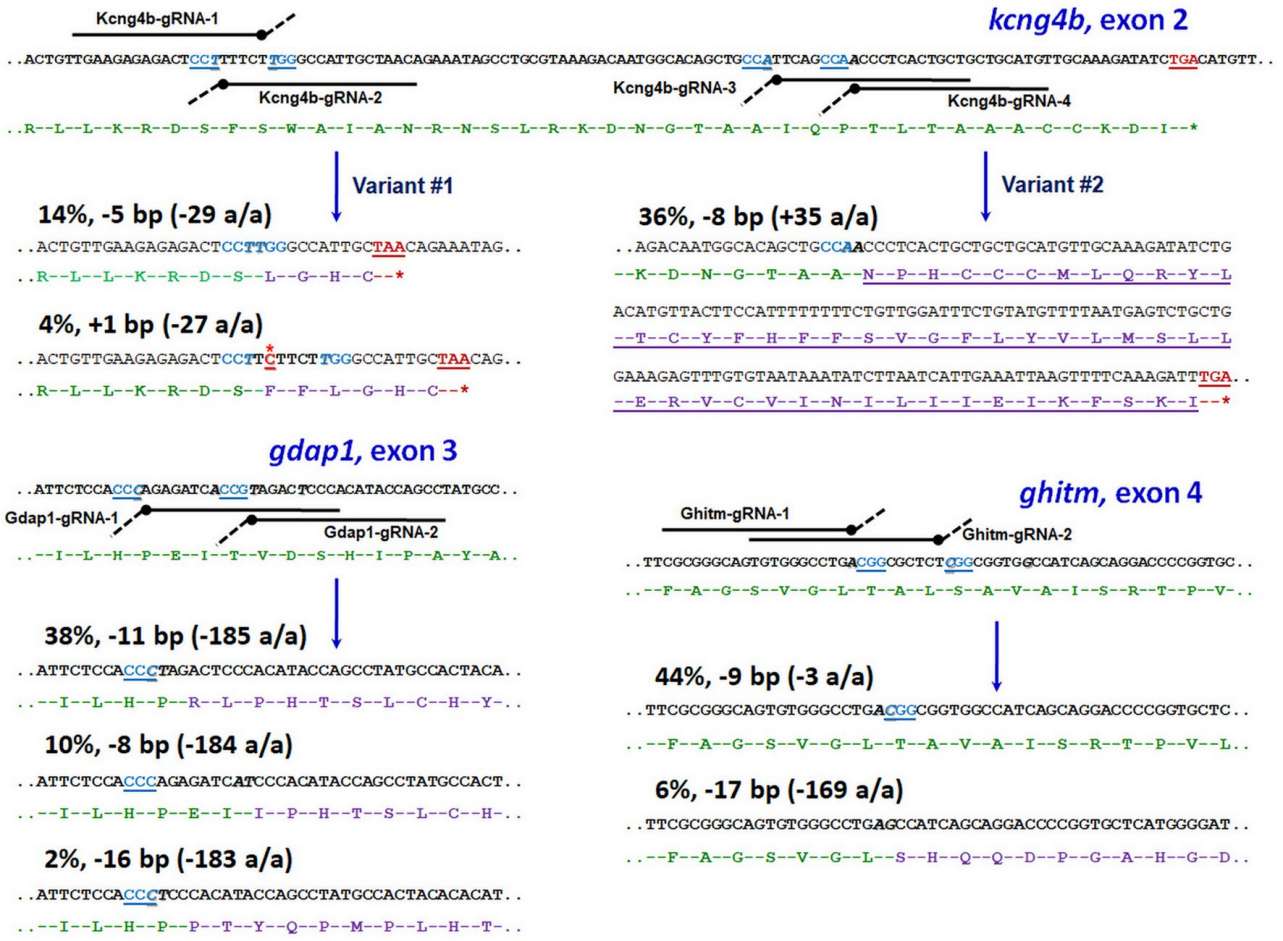
The schematic view of zebrafish (*D. rerio*) *kcng4b*, *gdap1* and *ghitm* mutant generation. Stop-codons are colored red, PAM-sequences are underlined and colored blue. Bold italics shows the DNA joining nucleotides after Cas9 brakes. A wild-type translation is colored green, modified one in the mutants—purple

We here describe an improved method of CRISPR-Cas9 genome editing to generate precise deletion mutant alleles of zebrafish *kcng4b*, *gdap1* and *ghitm* (Fig. 1). Our method is based on the injection of two (instead of one) complementary gRNAs closely delimiting the target site along with the purified Cas9 protein into one cell *D. rerio* embryos. We found that for the 5′-target site of *kcng4b* (i.e. variant #1) 7 out of 50 (14%) F0 founder fish carried the 5 bp deletion that matches the gap between the two gRNAs (Fig. 1). This probably represents a synergistic effect of the two gRNAs, Kcng4b-gRNA-1 and Kcng4b-gRNA-2. Two founders (4%) had a cytosine insertion at the site targeted by Kcng4b-gRNA-2. At the 3′-targeted site of *kcng4b* (i.e. variant #2), 18 of 50 F0 founders (36%) had a deletion of 8 bp also precisely corresponding to the gap between the two gRNAs (Fig. 1).

In the case of *gdap1* mutagenesis, 19 F0 fish (38%) had an 11 bp deletion corresponding to the synergistic action of the Gdap1-gRNA-1 and Gdap1-gRNA-2, while 6 fish (12%) exhibited either a 3′-shifted deletion in respect to Gdap1-gRNA-1 and Gdap1-gRNA-2 delimited site or a 16-bp deletion mediated by the Gdap1-gRNA-1 alone (Fig. 1).

For the *ghitm* the number of mutants with the precise deletion was 22 (44%), while 3 fish (6%) with different deletion mutations were detected (Fig. 1). The first group of mutants corresponds to the synergistic Ghitm-gRNA-1 and Ghitm-gRNA-2 action, whereas the second, minor group represents the result of CRISPR-Cas9 mutagenesis of the Ghitm-gRNA-1 site alone.

**Fig. 2:**
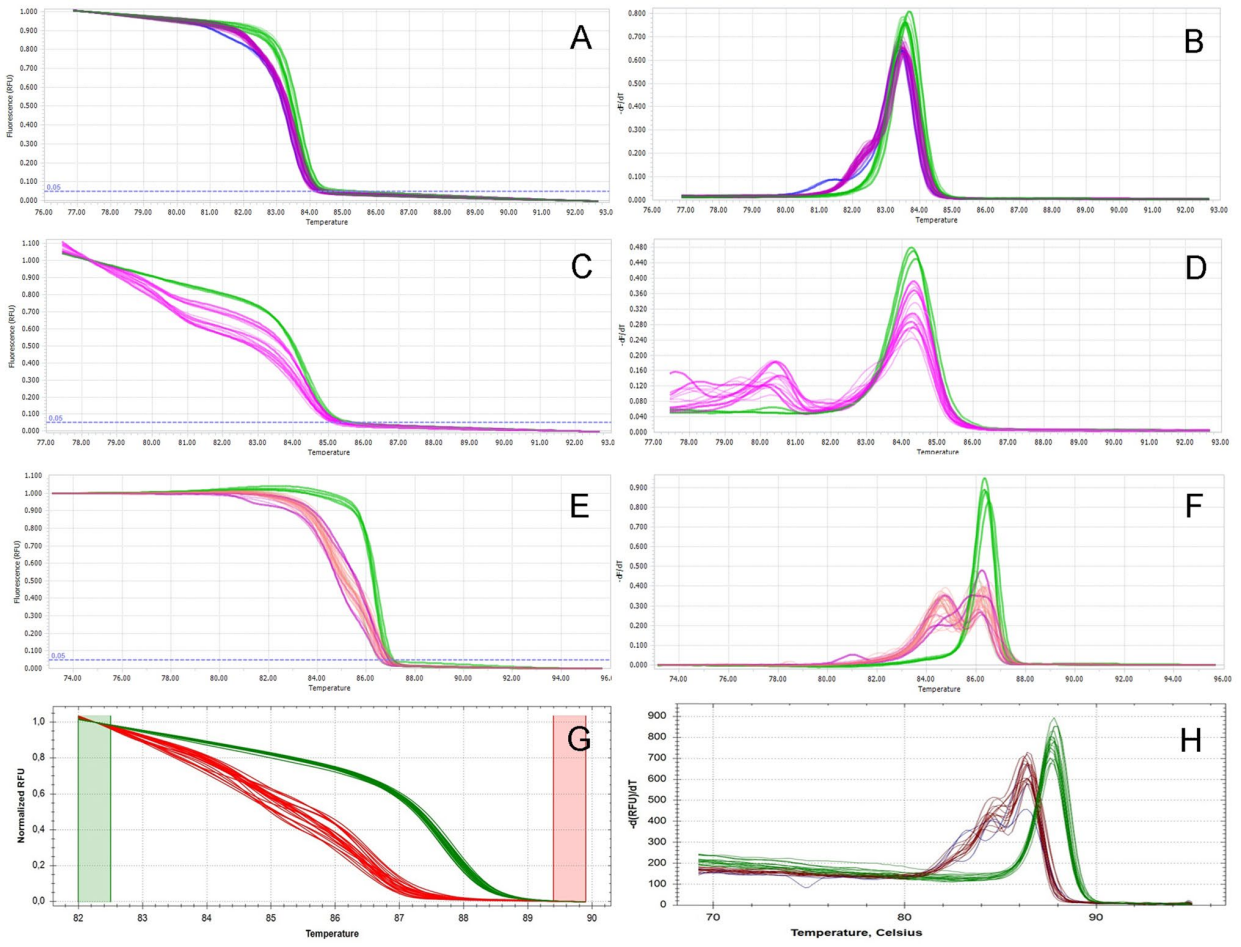
The HRM analysis of the fish generated by precise two gRNAs CRISPR-Cas9 mutagenesis. (**a**, **b**) *kcng4b* variant #1; (**c**, **d**) *kcng4b* variant #2; (**e**, **f**) *gdap1*; (**g**, **h**) *ghitm*. (**a**, **c**, **e**, **g**) Normalized melting curves; (**b**, **d**, **f**, **h**) normalized melting peaks. Green—wild type; purple, red and magenta—heterozygote precise deletion mutants; blue—minor mutant fractions

All F0 fish carrying the CRISPR-Cas9 modification were heterozygotes. HRM detected mutations (Fig. 2) and DNA sequencing confirmed their presence.

A follow-up analysis of F1 mutant in-cross offspring of *kcng4b* variant #1 showed the characteristic developmental defect of the *kcng4b* phenotype described earlier [13] for ~25% of embryos (Supplementary Fig. 1). In contrast, ~25% of homozygotes of the *kcng4b* variant #2 showed a developmental phenotype different from the one described by Shen et al. [13] (Supplementary Fig. 1), which could be explained by the fact that the mutation does not lead to a premature truncation (Fig. 1). All *kcng4b* mutants exhibiting the specific phenotype described above died during the first eight days of development. This further supports the observation that all F0 founders were heterozygotes. The HRM analysis showed that surviving F1 offspring were only heterozygotes and wild type animals (Fig. 2).

Around 25% of *gdap1* F0 mutant in-cross offspring exhibited a phenotype resembling the one observed after *gdap1* morpholino knock-down described previously [14] (Supplementary Fig. 1), while there was no obvious phenotype in *ghitm* mutant F1 offspring apparent in the first 5 days of development. An analysis of these *gdap1* and *ghitm* mutants’ survival beyond this time point was precluded by the limitations imposed by our ethical regulation protocol.

**Fig. 3:**
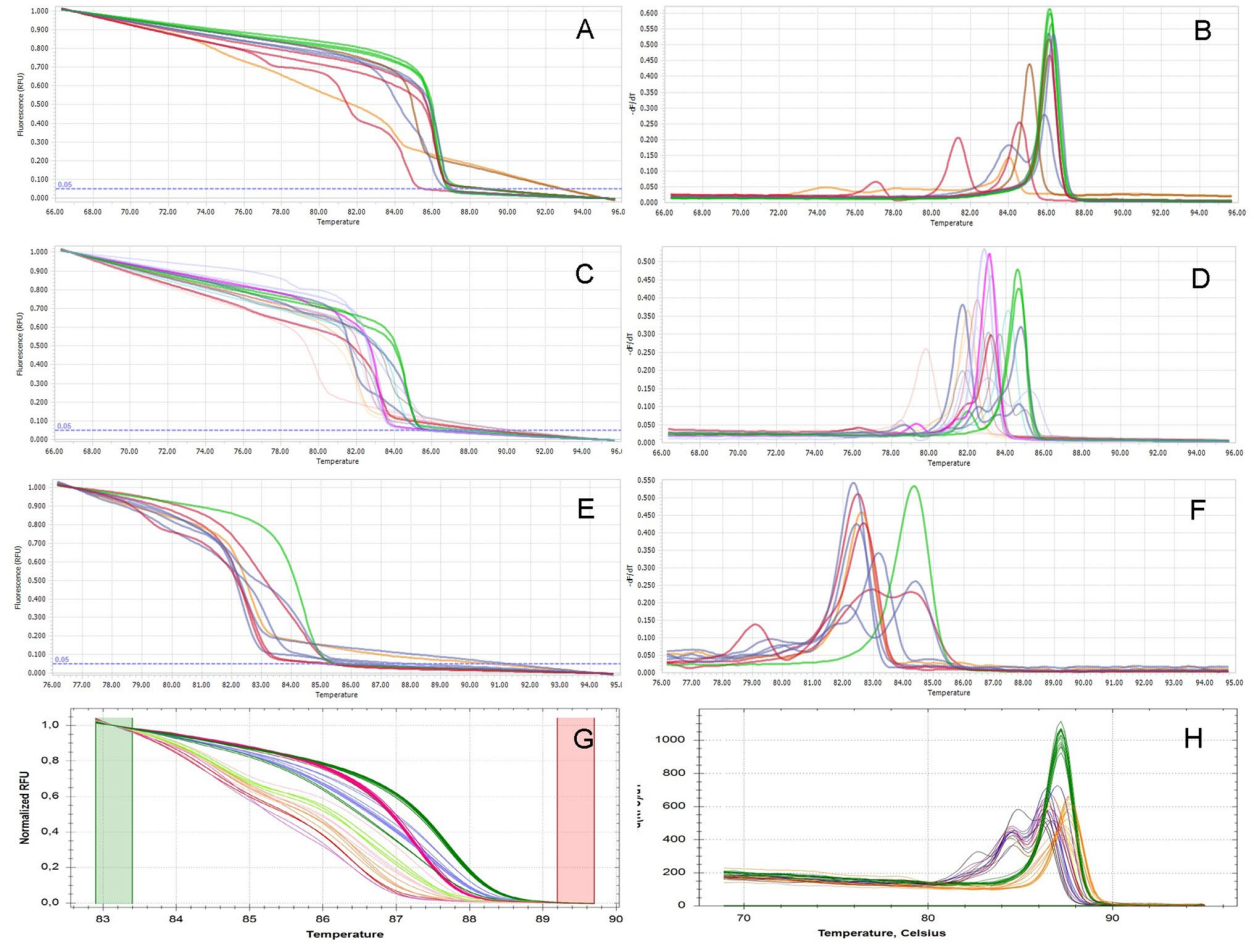
The HRM analysis of the fish generated by one gRNA CRISPR-Cas9 mutagenesis. (**a**, **b**) kcng4-gRNA-1; (**c**, **d**) kcng4b-gRNA-3; (**e**, **f**) Gdap1-gRNA-1; (**g**, **h**) Ghitm-gRNA-1. (**a**, **c**, **e**, **g**) Normalized melting curves; (**b**, **d**, **f**, **h**) normalized melting peaks. Green—wild type

In contrast to our new improved method using two gRNAs, the HRM analysis of the mutants of all three genes (*kcng4b*, *gdap1* and *ghitm*) generated by the traditional single gRNA CRISPR-Cas9 approach revealed a spectrum of variants. None of them represented the majority or a unified group (Fig. 3). This supports our claim that the use of two gRNAs creates more precise deletions.

## Discussion

Our modification of the CRISPR-Cas9 genome editing based on two complementary gRNAs that delimit the intervention site generates a precise deletion of several nucleotides in the target gene. It makes the outcome of mutagenesis more predictable, mutant screening much easier and allows the introduction of different open reading frame (ORF) shift mutations resulting in amino acid sequence changes, mainly preliminary truncation. The precise mutagenesis was confirmed for two different sites in the *kcng4b* gene, and single sites in the *gdap1* and *ghitm* genes, with a different number of nucleotides deleted, in a range from 5 to 11. This caused different ORF shifts, resulting in premature truncation in the *kcng4b* variant #1 and *gdap1*, no frame shift in the case of *ghitm*, and prolongation of the polypeptide in the *kcng4b* variant #2 (Fig. 1).

A rather negligible difference in the efficiency of generating precise deletion mutants was detected: 14% and 36% for *kcng4b* variant #1 and #2, 38% for *gdap1* and 44% for *ghitm*. These differences could be explained either by (1) specificity of gRNA action and peculiarities of the targeted site or (2) by mutant negative selection. The first is supported by the fact that the non-precise deletion mutants represent the minority. Moreover, the percentage of such incidental mutagenesis differs for all three genes: 4% for *kcng4b* variant #1 with lack in variant #2, 12% for *gdap1*, and 6% for *ghitm*. The *kcng4b* variant #1 incidental mutagenesis represents the insertion of a cytosine, which corresponds to the Kcng4b-gRNA-2 site-specific Cas9 action and follow-up DNA repair (Fig. 1). The *ghitm* incidental mutagenesis corresponds to cleavage at the Ghitm-gRNA-1 site. The same was detected for Gdap1-gRNA-1 (Fig. 1). All this suggests the unequal activity of the two gRNAs. Besides, the gRNA orientation may play a role. The more efficient *kcng4b* variant #2 mutagenesis, as well as the *gdap1* and *ghitm* ones seem to rely on correspondence of both gRNAs to the same DNA strand, whereas in the less efficient *kcng4b* variant #1 mutagenesis the gRNAs targeted opposite DNA strands (Fig. 1). Perhaps, different targeting of DNA strands may influence the effectiveness of Cas9 nuclease.

Only one insertion (*kcng4b* variant #1) was detected in our experiments. This supports the idea of a cooperative action of the gRNA pair even in cases of minor imprecise deletions. Presence of such incidental deletions may be caused by the DNA repair activity after a precise double gRNAs’ directed cleavage.

As to the mutant negative selection and its contribution to the effectiveness of mutagenesis, it is important to note that the survival rate of the two *kcng4b* variants was different. This may be caused by a toxic loss-of-function of the variant #1 mutation causing a reduction in survival even in the heterozygous state, e.g. through a deleterious effect of the mutation on spermatogenesis and/or survival of males as reported in rodents [15]. In support of a negative selection of mutants there is the highest efficiency of the *ghitm* mutagenesis (44%) where deletion did not lead to ORF shift.

## Conclusions

Our modified version of the CRISPR-Cas9 genome editing based on the two complementary gRNAs and Cas9 protein injection allows the precise deletion of several nucleotides in the target gene. HRM-based identification of mutants is an effective, rapid, economical and reliable tool for the selection of heterozygotes.

## Supplementary information

Below is the link to the electronic supplementary material.

Supplementary material 1 (JPG 264 kb)

## Data Availability

All data presented are available by request according the IIMCB Regulations.
