# Supplementary figures and images for: An improved method for precise genome editing in zebrafish using CRISPR-Cas9 technique
Source: Mol Biol Rep. 2021 Jan 22;48(2):1951–7. doi: 10.1007/s11033-020-06125-8 (PMC7925485; doi:10.1007/s11033-020-06125-8)

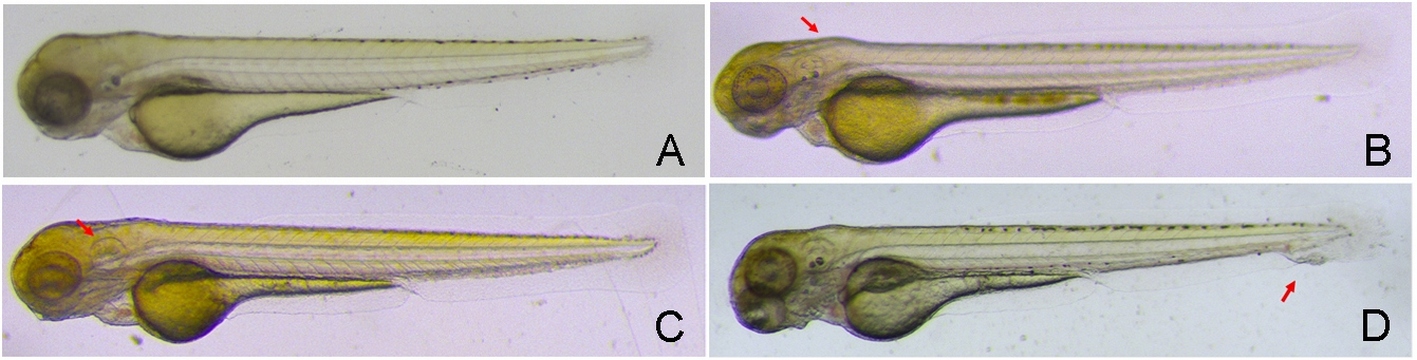

Supplement: Supplementary file 1 — Supplementary material 1 (JPG 264 kb) [file 11033_2020_6125_MOESM1_ESM.jpg]
